# Can primary care physician recommendation improve influenza vaccine uptake among older adults? A community health centre-based experimental study in China

**DOI:** 10.1186/s12875-023-01980-3

**Published:** 2023-01-17

**Authors:** Yating You, Xiaoheng Li, Shiqiang Jiang, Jing Liang, Pei Xie, Xuan Zou, Gang Liu, Xinxin Han

**Affiliations:** 1 School of Public Health and Emergency Management, Southern University of Science and Technology, Office 120, Taizhou Hall, 1088 Xueyuan Ave, Nanshan District, Shenzhen, China; 2 Shenzhen Centre for Disease Control and Prevention, 8 Longyuan Road, Nanshan District, Shenzhen, China

**Keywords:** Influenza vaccination, Older adults, Primary care physicians, Vaccination recommendation, Community health centres

## Abstract

**Background:**

To promote influenza vaccination coverage, a Chinese megacity, Shenzhen provides free influenza vaccination to its residents aged 60 years and above through community health centres (CHCs) since October 2016. A community health centre-based experiment was conducted by asking primary care physicians (PCPs) working in the intervention health centres to proactively recommend influenza vaccination to their patients aged 60 and above during their patients’ visits.

**Methods:**

This study used an experimental design and a survey design. The experimental design evaluated the effect of PCP recommendation on influenza vaccination. A total of 24 CHCs were randomly selected as the intervention (involving 3814 participants) and control (3072 participants) group evenly. The intervention study period was during the 2017–2018 flu season. The 2016–2017 flu season was considered as the baseline comparison. The survey design examined changes in knowledge, attitude, and practice of influenza vaccination among older participants before and after the free influenza vaccination implementation. We randomly invited 1200 participants aged 60 and above during their visits to CHCs in October 2016 and followed them up until October 2017; among them, 958 participants completed the follow-up survey using the same questionnaire.

**Results:**

In the 2017–2018 flu season, 1,100 more patients got vaccinated in the intervention group under PCP recommendation compared with the 2016–2017 flu season. Among the 958 older adults in the post-implementation period, 77.5% had heard about the influenza vaccine, which was 24.7% higher than in the pre-implementation period; 84.8% of participants were aware where to take influenza vaccines, with the most improvement of 37.2% among all knowledge related questions; 62.5% of them agreed that patients with chronic diseases should have influenza vaccine, which was 19.1% higher than those being surveyed before the implementation period. About 83.6% of participants agreed older adults should have influenza vaccine, but there were still 58.4% who considered themselves too healthy to get vaccinated.

**Conclusion:**

PCP recommendation improved influenza vaccine uptake and knowledge, attitude, and practice levels regarding influenza vaccination among older adults. More health policies and health education should be made to raise vaccination willingness and improve vaccination coverage among older adults.

## Background

Influenza is a highly-infected disease that can cause severe illnesses and even deaths [1]. Older adults, especially those with underlying diseases, are at a higher risk of being infected with or dying from influenza [2, 3]. Vaccination is the most effective way to protect against influenza [4]. Existing evidence suggests that influenza vaccine uptakes can reduce the severity level of physical conditions [5] and prevent influenza-related hospitalizations and deaths among older adults [6–8]. In developed countries, the rates of influenza vaccination among older adults are relatively high, for instance, 79.0% in the United Kingdom and 75.2% in the United States [9, 10].

In contrast, the coverage of influenza vaccination in China remains relatively low-only 3.8% of the older participants were vaccinated in a national survey study during the 2018–2019 flu season [11]. Prior studies indicated that provider-level barriers and a lack of willingness to take influenza vaccines among populations contributed to the low vaccination coverage in China [12, 13]. On one hand, the knowledge about influenza vaccination and the vaccination status of primary care physicians (PCPs) are important factors influencing receivers’ willingness to have influenza vaccines [14–16]. On the other hand, patients’ mistrust and physical violence towards physicians may affect PCPs’ willingness to recommend vaccine uptakes to the at-risk population in China [17].

In 2009, with the implementation of the national medical reform, China launched the national basic public health service program, which required primary healthcare facilities to deliver defined public health services, including vaccination services, physical check-ups, and health management to older residents. [18]. In urban areas, primary healthcare facilities are primarily community health centres (CHCs). Hence, widely-distributed community health centres are fully responsible for vaccination services to urban residents, including influenza vaccination to older adults [19]. People of all ages can take influenza vaccination at community health centres through making an appointment.

Evidence has shown that providing influenza vaccination free of charge might improve vaccination coverage [20]. To promote influenza vaccination coverage among older adults, some cities in China provide influenza vaccination services free of charge for adults aged 60 and above at CHCs [21]. Since October 2016, Shenzhen, an international tech hub as well as a mega-city in China with more than 0.94 million older adults, started to offer free influenza vaccination services to its residents aged 60 years and above through CHCs. Alongside this free influenza vaccination policy, Shenzhen Centres for Disease Prevention and Control also conducted a community health centre-based experiment. This experiment asked PCPs working in CHCs to proactively offer influenza vaccination recommendations to their older patients in their routine work. Choosing PCPs who work in CHCs to provide vaccination recommendations is because they are responsible for providing health management and follow-up services to older adults under the national basic public health program. Therefore, older patients may be more likely to accept and follow vaccination recommendations during their visits to PCPs in CHCs.

The goal of this study had two folds. First, this study aimed to evaluate whether PCP could improve influenza vaccination among older adults. Second, this study compared the knowledge, attitude, and practice level of influenza vaccination among older adults before and after the free influenza vaccination program implementation in Shenzhen, China. Findings from this study can provide valuable insights regarding health policies on influenza vaccine uptakes among older adults in other regions and contribute efforts to improving influenza vaccination coverage.

## Methods

### Study design and settings

This study used an experimental design and a survey design. The experimental design evaluated the effect of PCP recommendation on influenza vaccine uptakes among older participants. The survey design examined changes in knowledge, attitude, and practice of influenza vaccination among older participants before and after the free influenza vaccination policy was implemented.

In China, CHCs are responsible for providing vaccination services to urban residents. The study sites were the CHCs in Shenzhen, China. About 5.36% (0.94 million) of the population in Shenzhen are aged 60 years and above [22]. A total of 711 CHCs were located widely in 11 districts in Shenzhen. A random stratified sampling of CHCs was adopted to assign centres to the intervention and control groups at all stages. In the first stage, 4 districts (including Futian, Nanshan, Bao’an, and Longgang District) were selected; in the second stage, 6 health centres among the selected districts were randomly selected; in the third stage, 3 out of the 6 health centres in each district were assigned randomly into the intervention group and the rest 3 into the control group. Therefore, a total of 24 health centres in 4 districts were selected, among which half were assigned to the intervention group and the other half to the control group.

### Intervention

**Fig. 1:**
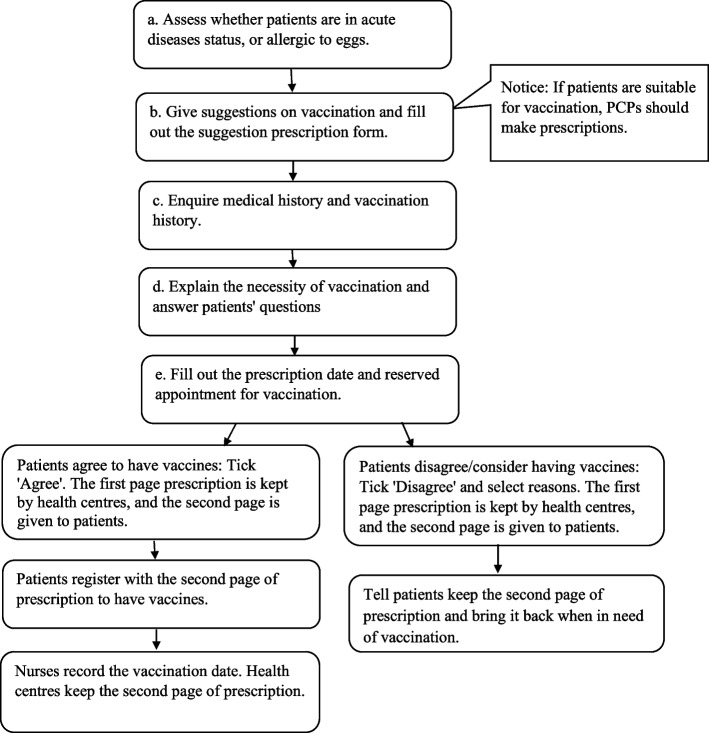
Flow chart of influenza vaccination recommendation routine by primary care physicians in community health centres in Shenzhen, China

In this experiment, PCPs working in the intervention health centres were asked to proactively recommend influenza vaccination to their patients who were aged 60 and above during their routine work and during their patients’ visits. PCPs working in the control health centres did not provide influenza vaccination recommendations for their patients. The intervention process is described as follows: when a patient visits an intervention health centre for any reason, PCPs would assess that patient’s health conditions and determine whether that patient is qualified to be vaccinated against influenza. If qualified, PCPs would make prescription forms, explain the necessity of vaccination and recommend that patient get vaccinated. If that patient agrees to be vaccinated, he/she will take the vaccine in the reserved time; if that patient does not agree to be vaccinated at that moment, he/she could keep the prescription forms in case they change their mind. The detailed standardized influenza vaccination recommendation procedure is shown in Fig. 1.

All PCPs from the intervention health centres received brief training about this standardized influenza vaccination recommendation process and were asked to offer influenza vaccination recommendations to their older patients in their daily work. PCPs from the control group did not receive such training. The intervention study period was during the 2017–2018 flu season (October 1, 2017 to April 30, 2018). The 2016–2017 flu season (October 1, 2016 to April 30, 2017) was considered as the baseline comparison. Older adults who: (1) were aged 60 years and above during the intervention; (2) had household registration and basic medical insurance; and (3) were qualified to have an extremely low risk of leading to severe health conditions based on medical history, were qualified for the free influenza vaccination program as the target of PCP recommendation.

### Questionnaire

We recruited participants aged 60 years and above and conducted a face-to-face survey interview during their visits to CHCs in October 2016 (pre-implementation of free influenza vaccination) and October 2017 (post-implementation of free influenza vaccination). Considering the survey feasibility in this megacity, Shenzhen, we randomly invited a total of 1200 older participants aged 60 and above during their visits to CHCs in October 2016 and followed them up until October 2017; among them, 958 participants completed the follow-up survey using the same questionnaire.

We used a self-designed questionnaire, which contained 11 questions related to knowledge, attitude, and practice of influenza vaccination. Three questions were about participants’ awareness of influenza vaccination. Participants were asked ‘Have you ever heard about influenza vaccination specifically offered to older adults?’, ‘Do you know about the free influenza vaccination program for older adults?’, and ‘Do you know where to get the influenza vaccine?’, with binary responses of ‘Yes’ and ‘No’.

The following section was about patients’ knowledge regarding influenza vaccination: (1) ‘Who has the priority to take influenza vaccines?’, with the following response options: ‘Healthcare workers’, ‘vulnerable populations and workers in care homes’, ‘students’, ‘older adults aged 60 years and above, 6-month to 5-year old children’, ‘patients with certain chronic diseases’, ‘carers and family members of under 6-month old babies’, and ‘women who are pregnant or ready to give birth during flu seasons’; (2) ‘How frequently should you be vaccinated?’, with three response options including ‘Once a year’, ‘Five times a year’, and ‘Do not know’; (3) ‘When is the suitable time to be vaccinated?’, with three options consisting of ‘In any season’, ‘Usually in autumn and winter’, and ‘Do not know’; (4) ‘How long is the protection duration of influenza vaccine?’, with three responses including ‘Six to eight months’, ‘At least five years’, and ‘Do not know’. Two questions were asked about older patients’ attitudes regarding influenza vaccination: ‘Whether you think older adults should have the influenza vaccine?’ and ‘Whether you think patients with chronic diseases should have the influenza vaccine?’, with responses of ‘Yes’, ‘No’ and ‘Do not know’.

Participants were then asked about their influenza vaccination history: ‘Ever vaccinated’ or ‘No’. If they reported ‘No’, they were asked to select the following reasons for not taking influenza vaccination: (1) ‘I am healthy enough and there is no need to take the vaccine’, (2) ‘I do not know about the influenza vaccination’, (3) ‘I think the influenza vaccine is not safe’, (4) ‘The influenza vaccine is not effective’, (5) ‘I have no time to get vaccinated’, (6) ‘Influenza is not a severe disease and it does not lead to any severe results’, (7) ‘It is inconvenient for me to get vaccinated’, (8) ‘It is too expensive to get vaccinated’, (9) ‘Physicians do not recommend to get the influenza vaccination’, and (10) ‘Other reasons’.

### Outcome measures

We evaluated two sets of outcomes. The primary outcomes were changes in the numbers of vaccinated older patients in the 24 studied health centres during the 2016–2017 flu season (pre-intervention of PCP recommendation: October 1, 2016 to April 30, 2017) and the 2017–2018 flu season (post-intervention of PCP recommendation: October 1, 2017 to April 30, 2018). The vaccination data were collected form the vaccination information record. The secondary outcomes were the older participants’ (1) knowledge of, attitude towards influenza vaccination; (2) vaccination status and reasons for not taking vaccination, which were collected from our self-designed questionnaire.

### Statistical analyses

Survey results were reported as frequencies and percentages. We calculated the differences in the number of vaccinated older participants between the intervention and control groups during the 2016–2017 flu season (baseline comparison) and the 2017–2018 flu season. The differences in knowledge, attitude, vaccination status, reasons for not taking vaccination, the information access to the influenza vaccination related knowledge among older participants before and after the implementation of free influenza vaccination program were assessed by Chi-square tests. A two-sided *P* value less than 0.05 was considered as statistically significant. SPSS version 17.0 was used for all analyses.

### Ethical consideration

Ethics approval was obtained from the Institutional Review Board of Shenzhen Centres for Disease Prevention and Control. Informed consent was obtained from all participated PCPs and patients in the study.

## Results

### Differences in the numbers of vaccinated older patients after PCP recommendation

In the intervention group, a total of 3814 patients received influenza vaccination. After PCPs in intervention groups proactively recommended their older patients to have influenza vaccine, 1100 more patients were vaccinated in the 2017–2018 flu season. Moreover, in the control group, 3072 patients received influenza vaccination, whereas after PCP recommendation, even 86 fewer got vaccinated, compared to the baseline 2016–2017 flu season (Table 1). Under PCP recommendation, the number of vaccinated patients in intervention health centres increased most with the number of 453 in Futian District, followed by Nanshan District (diff. = 345), Bao’an District (diff. = 163) and Longgang District (diff. = 139). In contrast, in control health centres, the numbers of vaccinated patients decreased, with the number of 35 patients as the most in Longgang District, followed by Bao’an District (diff. = 31), Futian District (diff. = 12), and Nanshan District (diff. = 8) respectively.

**Table 1:** Differences in the numbers of vaccinated older patients in intervention and control health centres after PCP recommendation in Shenzhen, China

District	Health Centres	Number of Vaccinated Older Patients		Differences
		**Before PCP Recommendation (October 1,2016 to April 30, 2017)**	**After PCP Recommendation (October 1, 2017 to April 30,2018)**	
Total	Intervention (*N* = 12)	1357	2457	1100
	Control(*N* = 12)	1579	1493	-86
Futian	Intervention (*N* = 3)	342	795	453
	Control (*N* = 3)	511	499	-12
Nanshan	Intervention (*N* = 3)	262	607	345
	Control (*N* = 3)	368	360	-8
Longgang	Intervention (*N* = 3)	315	454	139
	Control (*N* = 3)	399	364	-35
Bao’an	Intervention (*N* = 3)	438	601	163
	Control (*N* = 3)	301	270	-31

### Differences in knowledge regarding influenza vaccination among older participants

After the one-year implementation of the free influenza vaccination program, 77.5% of older participants were aware of the influenza vaccine, which was 24.7% higher than that in the pre-implementation period (Table 2). Before the free vaccination was offered, 40.4% of participants knew the free influenza vaccination programme for older adults; after the free vaccination was offered, the proportion of older participants knowing the free influenza vaccination program increased by 21.8% significantly. Older participants improved their knowledge regarding influenza vaccination significantly in the following five items after the implementation of free vaccination program: 40.3% knew the prioritized populations of being vaccinated correctly (18.5% higher than that the pre-implementation period); 40.8% of them were aware of the frequency of influenza vaccination (18.0% higher than the pre-implementation period); 33.8% of them answered the most suitable time to be vaccinated correctly (14.2% higher than the pre-implementation period); 33.2% knew the best protection duration (21.6% higher than the pre-implementation period); 84.8% of participants were aware of the places to get influenza vaccine, which had the biggest improvement of 37.2% among all questions.

**Table 2:** Differences in knowledge regarding influenza vaccine uptakes among older participants before and after the free influenza vaccination policy implementation in Shenzhen, China

Characteristic	Pre-implementation^h^*N* = 1200	Post-implementation^h^*N* = 958	Diff. (%)
	**N (%)**	**N (%)**	
Awareness of influenza vaccine uptakes among older participants^a^	634(52.8)	742(77.5)	24.7
Awareness of free influenza vaccination program for older participants^b^	485(40.4)	596(62.2)	21.8
Ever know about influenza vaccination site^c^	571(47.6)	812(84.8)	37.2
Correct knowledge of priority vaccination populations^d^	262(21.8)	386(40.3)	18.5
Correct knowledge of vaccination frequency^e^	273(22.8)	395(40.8)	18.0
Correct knowledge of the most suitable time to be vaccinated^f^	235(19.6)	324(33.8)	14.2
Correct knowledge of the vaccination protection duration^g^	139(11.6)	318(33.2)	21.6

*P* values were calculated from Chi-square tests and group comparisons of percentages of all questions were less than 0.05

^a^ Participants who answered they have ever heard about influenza vaccine uptakes among older participants were considered that they have the awareness but did not indicate correct knowledge of influenza vaccination offered to older adults

^b^ Participants who answered they have ever heard about the free influenza vaccination program for older adults were considered to have the awareness but did not indicate correct knowledge of the free influenza vaccination program

^c^ Participants who answered they have known about influenza vaccination site were considered to have the awareness but did not indicate correct knowledge of influenza vaccination site

^d^ Participants who selected following answers: ‘Healthcare workers’, ‘vulnerable populations and workers in care homes’, ‘students’, ‘older adults aged 60 years and above, 6-month to 5-year-old children’, ‘patients with certain chronic diseases’, ‘carers and family members of under 6-month-old babies’, and ‘women who are pregnant or ready to give birth during flu seasons’, were considered to have the correct knowledge of the prioritised populations of being vaccinated

^e^ Participants who answered ‘Once a year’ were considered to have the correct knowledge of the vaccination frequency

^f^ Participants who answered ‘Usually in autumn and winter’ were considered to have the correct knowledge of suitable time to be vaccinated

^g^ Participants who answered ‘Six to eight months’ were considered to have the correct knowledge of vaccination protection duration

^h^ Pre-implementation means prior the city-wide free influenza vaccination for older adults program was implemented. Post-implementation means after the city-wide free influenza vaccination for older adults program was implemented. Shenzhen implemented a city-wide free influenza vaccination for older adults program since October 2016

### Differences in attitude regarding influenza vaccination among older participants

Before the free vaccination program was available, 68.5% of participants agreed that older adults should have vaccine to prevent existing conditions from worsening (Table 3). After the implementation of the free vaccination program, the proportion of participants who regarded a must to have the influenza vaccine increased significantly to 83.6%. Meanwhile, 62.5% of them thought patients with chronic diseases should have the influenza vaccine uptakes, which was 19.1% higher than the pre-implementation period. It has indicated a statistically significant improvement (*P* < 0.05) during the post-implementation period.

**Table 3:** Differences in attitudes regarding influenza vaccination among older participants before and after the free influenza vaccination policy implementation in Shenzhen, China

Characteristic	Pre-implementation^c^*N* = 1200	Post-implementation^c^*N* = 958	Diff. (%)
	**N (%)**	**N (%)**	
Attitude towards older adults to take influenza vaccine^a^	822(68.5)	801(83.6)	15.1
Attitude towards patients with chronic diseases to have influenza vaccine^b^	521(43.4)	599(62.5)	19.1

*P* values were calculated from Chi-square tests and group comparisons of percentages of all questions were less than 0.05

^a^ Participants who thought ‘Older adults should have the influenza vaccine’ were considered to show the positive attitude towards influenza vaccination

^b^ Participants who thought ‘Patients with chronic diseases should have the influenza vaccine’ were considered to show the positive attitude towards influenza vaccination

^c^ Pre-implementation means prior the city-wide free influenza vaccination for older adults program was implemented. Post-implementation means after the city-wide free influenza vaccination for older adults program was implemented. Shenzhen implemented a city-wide free influenza vaccination for older adults program since October 2016

### Differences in influenza vaccination status and the top three reasons for not having vaccines

Before the free vaccination program was available, 66.8% participants were not aware of the influenza vaccination (Table 4). After the implementation of the free vaccination policy, the proportion of not knowing about the influenza vaccination decreased by 32.6% significantly. The most surprising finding was that 83.6% of participants considered it absolutely necessary to have the influenza vaccine for older adults, but as for themselves, 58.4% regarded it unnecessary due to their self-awareness of having a good health condition. Moreover, during the post-implementation period, 58.4% of participants thought they were healthy enough and there was no need to have influenza vaccine, 32.6% higher significantly than the pre-implementation. 22.7% participants still suggested they were not recommended influenza vaccination by their physicians when attending health centres in person, even with the active promotion of implementation of the free vaccination program, which was 19.1% higher than the pre-implementation period significantly.

**Table 4:** Differences in influenza vaccination status among older participants before and after the free influenza vaccination policy was implemented in Shenzhen, China and top three reasons for not having vaccines

Items	Pre-implementation^a^*N* = 1200	Post-implementation^a^*N* = 958	Diff. (%)
	**N (%)**	**N (%)**	
**Vaccination Status**			
Ever vaccinated	138(11.5)	358(37.4)	25.9
Vaccinated last year	45(3.8)	321(33.5)	29.7
**Top Three Reasons for Not Having Vaccines**			
Do not know about the influenza vaccination	801(66.8)	328(34.2)	-32.6
Healthy enough and there is no need to be vaccinated	309(25.8)	559(58.4)	32.6
Physicians do not recommend getting the influenza vaccination	43(3.6)	218(22.7)	19.1

*P* values were calculated from Chi-square tests and group comparisons of percentages of all questions were less than 0.05

^a^ Pre-implementation means prior the city-wide free influenza vaccination for older adults program was implemented. Post-implementation means after the city-wide free influenza vaccination for older adults program was implemented. Shenzhen implemented a city-wide free influenza vaccination for older adults program since October 2016

### Information access about influenza vaccination

**Fig. 2:**
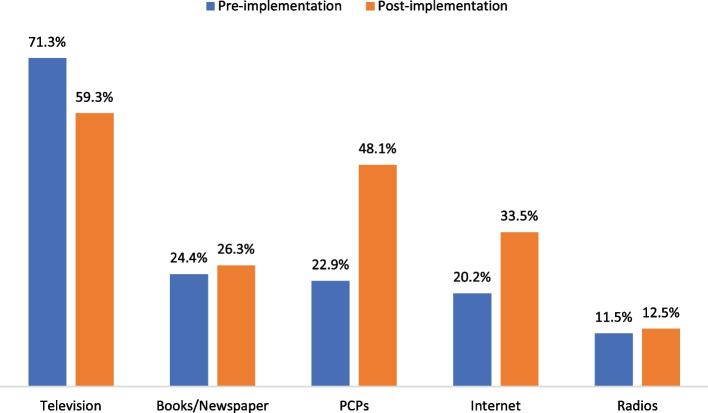
Information access about influenza vaccination among older participants. Note: Pre-implementation means prior the city-wide free influenza vaccination for older adults program was implemented. Post-implementation means after the city-wide free influenza vaccination for older adults program was implemented. Shenzhen implemented a city-wide free influenza vaccination for older adults program since October 2016

Television was the most frequently used access for older participants to obtain knowledge about influenza vaccination. Meanwhile, older participants were more likely to obtain knowledge from PCPs and the internet with improvements of 25.2% and 13.3% respectively, after the free vaccination program was implemented (Fig. 2).

## Discussion

Our study provided evidence that PCP recommendation was as an effective intervention in increasing the numbers of influenza vaccine uptake and improving the knowledge, attitude, and practice level regarding influenza vaccination among older adults, with the implementation of free influenza vaccination program. It also highlighted the importance of incorporating PCP vaccination recommendation for older adults in routine work in CHCs.

Our findings were consistent with prior results indicating that healthcare professionals, especially primary care physicians, played an important role in improving influenza coverage among diverse populations [23, 24]. In a previous study, approximately 78% of older adults showed their willingness to take influenza vaccine if being recommended by a physician [15]. However, in a previous situation in Shenzhen, where only community nurses assessed patients’ health status and provided vaccine uptakes directly, PCPs were not directly involved in any step of vaccination services, because recommendations of influenza vaccination were not incorporated in their daily work routines. Hence, patients’ lack of primary suggestions from PCPs on influenza vaccination might be an important barrier in promoting influenza coverage among older adults. Evidence showed that enhancements in professional knowledge in influenza vaccination among PCPs might increase the possibility of vaccination recommendations [25, 26]. The CHCs should provide regular relevant training including delivering knowledge on health education, assessment methods, and recommendations of influenza vaccine uptakes for PCPs. After revising their work routines and obtaining the latest knowledge on vaccines, PCPs know influenza vaccination better objectively and are more likely to follow the instruction of standardised routine to recommend vaccine uptakes to older adults, which acts as the basis of PCP recommendation. PCP recommendation should be included in their daily work routine and CHCs also need to provide opportunities for PCPs to communicate with and deliver this knowledge to their patients.

Although we found that knowledge and attitude regarding influenza vaccine uptakes among older adults were improved after the implementation of free influenza vaccination program, the willingness of patients to take influenza vaccination remained still relatively low [20]. This calls for more policy actions to be conducted in a wider range and more health education efforts should be made in improving the willingness of patients to have influenza vaccine. The free influenza vaccination program might promote influenza vaccine uptake among older adults aged 60 years and above as it is free of charge and incentive for target populations in Shenzhen, China. Since PCP recommendation has been proved effective in improving the numbers of influenza vaccine uptakes, the integration of free vaccination program and PCP recommendation does play an important role in improving knowledge, attitude, and practice regarding influenza vaccination, and increasing the numbers of influenza vaccine uptakes among older adults in the future.

Under the progress of free vaccination program, people started to put more emphases on the influenza prevention intervention measures. In China, PCPs in CHCs were considered as one of the most fundamental healthcare workers to consult with, where people obtained most of their health education related knowledge. According to our results, older patients showed increased willingness to access their vaccination knowledge from PCPs and the internet. Growing interest has shown in the role of internet in public health promotion due to increasing people’s health enquiries on the internet and social media [27]. However, harmful misinformation spreads across networks, including anti-vaccination events, which deepens vaccine hesitancy and misunderstanding [28]. Future intervention could be considered to add more health education about influenza and influenza vaccination in public for older adults not only through PCPs routine, but also websites and social medias. The free influenza vaccination program has been proved effective to improve patients’ willingness to take influenza to some extent, which is consistent with previous research [20]. The effect of integrating influenza vaccination recommendation into the routine work of PCPs combined with free vaccination programs merits verifications in national ranges, but health policies in each region should be developed with features by relevant departments.

Our study has several limitations. First, recall bias might occur in this process as we used a self-reported questionnaire to investigate patients’ knowledge of influenza vaccine uptake. Second, it should be careful when generalizing our results to other cities in China, as Shenzhen is a mega-city, but only 5.36% of its population are older adults [22], which is far below the national average (18.7%) [29]. Third, due to data limitation, some information such as patient characteristics were not available. Further research could be conducted to explore and evaluate more effective interventions to increase influenza vaccination coverage among other at-risk populations.

## Conclusions

With the implementation of free influenza vaccination program in Shenzhen, China, PCP recommendation can improve older adults’ influenza vaccine uptake and people’s knowledge, attitude, and practice level towards influenza and influenza vaccination in Shenzhen, China. PCPs should recommend influenza vaccine uptakes to the older populations as their work routine in every health centre in China to improve vaccination coverage. Additionally, the free influenza vaccine uptake programs should be promoted among all older adults in China.

## Data Availability

The access to the data that support the findings of this study is available through emailing the corresponding author upon reasonable request.
